# Supplementary material for: Improving scheduling performance in congested networks
Source: PeerJ Comput Sci. 2021 Nov 1;7:e754. doi: 10.7717/peerj-cs.754 (PMC8576556; doi:10.7717/peerj-cs.754)
Supplement: Supplemental Information 1 [file peerj-cs-07-754-s001.docx]

**Model Source Application Header File**

/*

Author: Arif Husen

File: ibps.h

Written: 15/05/2016

Modifed: 15/05/2016

*/

#include "timer-handler.h"

#include "packet.h"

#include "app.h"

#include "udp-ms.h"

#include <vector>

#include <string>

#include <sstream>

// This is used for receiver's received packet accounting

struct pkt_accounting {

int last_seq; // sequence number of last received MM pkt

int last_scale; // rate (0-4) of last acked

int lost_pkts; // number of lost pkts since last ack

int recv_pkts; // number of received pkts since last ack

double rtt; // round trip time

double last_ts;

};

class MsApp;

// Sender uses this timer to

// schedule next app data packet transmission time

class SendTimer : public TimerHandler {

public:

SendTimer(MsApp* t) : TimerHandler(), t_(t) {}

inline virtual void expire(Event*);

protected:

MsApp* t_;

};

// Reciver uses this timer to schedule

// next ack packet transmission time

class AckTimer : public TimerHandler {

public:

AckTimer(MsApp* t) : TimerHandler(), t_(t) {}

inline virtual void expire(Event*);

protected:

MsApp* t_;

};

// Mulitmedia Application Class Definition

class MsApp : public Application {

public:

MsApp();

void send_ms_pkt(); // called by SendTimer:expire (Sender)

void send_ack_pkt(); // called by AckTimer:expire (Receiver)

//int p = 1;

int profile[6][25];

int BP[6][25];

int PS[6][25];

protected:

int command(int argc, const char*const* argv);

void start(); // Start sending data packets (Sender)

void stop(); // Stop sending data packets (Sender)

private:

void init();

inline double next_snd_time(); // (Sender)

virtual void recv_msg(int nbytes, const char *msg = 0); // (Sender/Receiver)

void set_scale(const hdr_ms *mh_buf); // (Sender)

void adjust_scale(void); // (Receiver)

void account_recv_pkt(const hdr_ms *mh_buf); // (Receiver)

void init_recv_pkt_accounting(); // (Receiver)

double getRate(int bp);

int load_ti();

int load_bp();

int load_ps();

int get_pktSize(int bp,int prof);

int get_bp();

double rate[5]; // Transmission rates associated to scale values

double interval_; // Application data packet transmission interval

// level of the node sending the packets, could be 1 = access, 2 = aggregation, 3= core

int pktsize_; // Application data packet size

//TclObject** test_;

int trafprof_;

int nodelevel_;

int random_; // If 1 add randomness to the interval

int running_; // If 1 application is running

int seq_; // Application data packet sequence number

int scale_; // Media scale parameter

//double TFI[5];

double BPS[5];

int USERS_;

double SRATE_;

pkt_accounting p_accnt;

SendTimer snd_timer_; // SendTimer

AckTimer ack_timer_; // AckTimer

};

**Model Source Implementation File**

/*

Author: Arif Husen , VU, MSCS Thesis Research

File: ibps.h

Written: 15/05/2016

Modifed: 15/05/2016

*/

#include "random.h"

#include "ms-app.h"

#include <iostream>

#include "tcl.h"

#include "tclcl.h"

#include <string>

#include <sstream>

#include <fstream>

#include <stdlib.h>

// Modelsource OTcl linkage class

static class MsAppClass : public TclClass {

public:

MsAppClass() : TclClass("Application/MsApp") {}

TclObject* create(int, const char*const*) {

return (new MsApp);

}

} class_app_ms;

// When snd_timer_ expires call Modelsource:send_mm_pkt()

void SendTimer::expire(Event*)

{

t_->send_ms_pkt();

}

// When ack_timer_ expires call Modelsource:send_ack_pkt()

void AckTimer::expire(Event*)

{

t_->send_ack_pkt();

}

// Constructor (also initialize instances of timers)

MsApp::MsApp() : running_(0), snd_timer_(this), ack_timer_(this)

{

//bind("pktsize_", &pktsize_);

bind("trafprof_", &trafprof_);

bind("nodelevel_",&nodelevel_);

bind_bool("random_", &random_);

load_ti();

load_bp();

load_ps();

pktsize_ = get_pktSize(get_bp(),trafprof_); //initial packet size is bp = 0

}

double MsApp::getRate(int bp) {

double r = 0;

//double BP6[5] = {0,2,4,6,8};

//double profile4[5] = {};

//double profile4[5] = {};

//double profile4[5] = {};

switch (trafprof_) {

case 0:

rate[0] = profile[0][0]*1000*1000;

rate[1] = profile[0][1]*1000*1000;

rate[2] = profile[0][2]*1000*1000;

rate[3] = profile[0][3]*1000*1000;

rate[4] = profile[0][4]*1000*1000;

rate[5] = profile[0][5]*1000*1000;

BPS[0] = BP[0][0];

BPS[1] = BP[0][1];

BPS[2] = BP[0][2];

BPS[3] = BP[0][3];

BPS[4] = BP[0][4];

r = profile[0][bp]*1000*1000;

//cout << (int)trafprof_ << endl;

break;

case 1:

rate[0] = profile[1][0]*1000*1000;

rate[1] = profile[1][1]*1000*1000;

rate[2] = profile[1][2]*1000*1000;

rate[3] = profile[1][3]*1000*1000;

rate[4] = profile[1][4]*1000*1000;

rate[5] = profile[1][5]*1000*1000;

BPS[0] = BP[1][0];

BPS[1] = BP[1][1];

BPS[2] = BP[1][2];

BPS[3] = BP[1][3];

BPS[4] = BP[1][4];

r = profile[1][bp]*1000*1000;

//cout << (int)trafprof_ << " -" << rate[0] << "- " << rate[1] << endl;

break;

case 2:

rate[0] = profile[2][0]*1000*1000;

rate[1] = profile[2][1]*1000*1000;

rate[2] = profile[2][2]*1000*1000;

rate[3] = profile[2][3]*1000*1000;

rate[4] = profile[2][4]*1000*1000;

rate[5] = profile[2][5]*1000*1000;

BPS[0] = BP[2][0];

BPS[1] = BP[2][1];

BPS[2] = BP[2][2];

BPS[3] = BP[2][3];

BPS[4] = BP[2][4];

r = profile[2][bp]*1000*1000;

//cout << (int)trafprof_ << endl;

break;

case 3:

rate[0] = profile[3][0]*1000*1000;

rate[1] = profile[3][1]*1000*1000;

rate[2] = profile[3][2]*1000*1000;

rate[3] = profile[3][3]*1000*1000;

rate[4] = profile[3][4]*1000*1000;

rate[5] = profile[3][5]*1000*1000;

BPS[0] = BP[3][0];

BPS[1] = BP[3][1];

BPS[2] = BP[3][2];

BPS[3] = BP[3][3];

BPS[4] = BP[3][4];

r = profile[3][bp]*1000*1000;

//cout << (int)trafprof_ << endl;

break;

case 4:

rate[0] = profile[4][0]*1000*1000;

rate[1] = profile[4][1]*1000*1000;

rate[2] = profile[4][2]*1000*1000;

rate[3] = profile[4][3]*1000*1000;

rate[4] = profile[4][4]*1000*1000;

rate[5] = profile[4][5]*1000*1000;

BPS[0] = BP[4][0];

BPS[1] = BP[4][1];

BPS[2] = BP[4][2];

BPS[3] = BP[4][3];

BPS[4] = BP[4][4];

r = profile[4][bp]*1000*1000;

//cout << (int)trafprof_ << endl;

break;

case 5:

rate[0] = profile[5][0]*1000*1000;

rate[1] = profile[5][1]*1000*1000;

rate[2] = profile[5][2]*1000*1000;

rate[3] = profile[5][3]*1000*1000;

rate[4] = profile[5][4]*1000*1000;

rate[5] = profile[5][5]*1000*1000;

BPS[0] = BP[5][0];

BPS[1] = BP[5][1];

BPS[2] = BP[5][2];

BPS[3] = BP[5][3];

BPS[4] = BP[5][4];

r = profile[5][bp]*1000*1000;

//cout << (int)trafprof_ << endl;

break;

}

return r;

}

// OTcl command interpreter

int MsApp::command(int argc, const char*const* argv)

{

Tcl& tcl = Tcl::instance();

if (argc == 3) {

if (strcmp(argv[1], "attach-agent") == 0) {

agent_ = (Agent*) TclObject::lookup(argv[2]);

if (agent_ == 0) {

tcl.resultf("no such agent %s", argv[2]);

return(TCL_ERROR);

}

// Make sure the underlying agent support MM

tcl.resultf(" the supportMS = " , agent_->supportMS());

if(agent_->supportMS()) {

agent_->enableMS();

}

else {

tcl.resultf("agent \"%s\" does not support Model Source Application", argv[2]);

return(TCL_ERROR);

}

agent_->attachApp(this);

return(TCL_OK);

}

}

return (Application::command(argc, argv));

}

void MsApp::init()

{

scale_ = 0; // Start at minimum rate

seq_ = 0; // MM sequence number (start from 0)

pktsize_ = get_pktSize(get_bp(),trafprof_); // get initial packet size to bp = 0

interval_ = (double)(pktsize_ << 3)/(double)getRate(scale_);

}

void MsApp::start()

{

init();

running_ = 1;

send_ms_pkt();

}

void MsApp::stop()

{

running_ = 0;

}

// Send application data packet

void MsApp::send_ms_pkt()

{

hdr_ms mh_buf;

if (running_) {

// the below info is passed to UDPmm agent, which will write it

// to MM header after packet creation.

mh_buf.ack = 0; // This is a MM packet

mh_buf.seq = seq_++; // MM sequece number

mh_buf.nbytes = get_pktSize(get_bp(),trafprof_); // Size of MM packet (NOT UDP packet size)

mh_buf.time = Scheduler::instance().clock(); // Current time

mh_buf.scale = scale_; // Current scale value

agent_->sendmsg(get_pktSize(get_bp(),trafprof_), (char*) &mh_buf); // send to UDP

// Reschedule the send_pkt timer

double next_time_ = next_snd_time();

if(next_time_ > 0) snd_timer_.resched(next_time_);

}

}

// Schedule next data packet transmission time

double MsApp::next_snd_time()

{

// Recompute interval in case rate or size chages

int bp = get_bp();

pktsize_ = get_pktSize(bp,trafprof_);

interval_ = (double)(pktsize_ << 3)/(double)getRate(scale_);

double next_time_ = interval_;

//if(random_)

//next_time_ += interval_ * Random::uniform(-0.5, 0.5);

return next_time_;

}

// Receive message from underlying agent

void MsApp::recv_msg(int nbytes, const char *msg)

{

if(msg) {

hdr_ms* mh_buf = (hdr_ms*) msg;

if(mh_buf->ack == 1) {

// If received packet is ACK packet

//cout << "Ack Received " << endl;

set_scale(mh_buf);

}

else {

// If received packet is MM packet

account_recv_pkt(mh_buf);

//cout << "seq no is " << mh_buf->ack << endl;

if(mh_buf->seq == 0) send_ack_pkt();

}

}

}

// Sender sets its scale to what reciver notifies

void MsApp::set_scale(const hdr_ms *mh_buf)

{

scale_ = mh_buf->scale;

//cout << "new scale set ...." << scale_ << " rate is " << getRate(scale_)<< endl;

}

void MsApp::account_recv_pkt(const hdr_ms *mh_buf)

{

double local_time = Scheduler::instance().clock();

// Calculate RTT

if(mh_buf->seq == 0) {

init_recv_pkt_accounting();

p_accnt.rtt = 2*(local_time - mh_buf->time);

}

else

p_accnt.rtt = 0.9 * p_accnt.rtt + 0.1 * 2*(local_time - mh_buf->time);

// Count Received packets and Calculate Packet Loss

p_accnt.recv_pkts ++;

p_accnt.lost_pkts += (mh_buf->seq - p_accnt.last_seq - 1);

p_accnt.last_seq = mh_buf->seq;

p_accnt.last_ts = mh_buf->time;

}

void MsApp::init_recv_pkt_accounting()

{

p_accnt.last_seq = -1;

p_accnt.last_scale = 0;

p_accnt.lost_pkts = 0;

p_accnt.recv_pkts = 0;

}

void MsApp::send_ack_pkt(void)

{

double local_time = Scheduler::instance().clock();

adjust_scale();

// send ack message

hdr_ms ack_buf;

ack_buf.ack = 1; // this packet is ack packet

ack_buf.time = local_time;

ack_buf.nbytes = 40; // Ack packet size is 40 Bytes

ack_buf.scale = p_accnt.last_scale;

agent_->sendmsg(ack_buf.nbytes, (char*) &ack_buf);

//cout << " ACK Send " << p_accnt.rtt << endl;

// schedul next ACK time

ack_timer_.resched(p_accnt.rtt);

}

void MsApp::adjust_scale(void)

{

if(p_accnt.recv_pkts > 0) {

if (p_accnt.last_ts > BPS[0] && p_accnt.last_ts <= BPS[1]) { p_accnt.last_scale = 0;}

if (p_accnt.last_ts > BPS[1] && p_accnt.last_ts <= BPS[2]) { p_accnt.last_scale = 1;}

if (p_accnt.last_ts > BPS[2] && p_accnt.last_ts <= BPS[3]) { p_accnt.last_scale = 2;}

if (p_accnt.last_ts > BPS[3] && p_accnt.last_ts <= BPS[4]) { p_accnt.last_scale = 3;}

if (p_accnt.last_ts > BPS[4]) { p_accnt.last_scale = 4;}

}

p_accnt.recv_pkts = 0;

p_accnt.lost_pkts = 0;

}

int MsApp::load_ti(){

int status = 0;

ifstream infile;

infile.open ("/home/arifhusen/ns2/ns-2.35/tcl/msprofiles/ti.dat", ifstream::in);

string str;

string tix;

int line = 0;

if (infile.is_open()) {

while (getline(infile, str))

{

//cout << str << endl;

int i=0;

stringstream ssin(str);

while(ssin.good() && i <= 24){

ssin >> tix;

int b = atoi(tix.c_str());

profile[line][i] = b; // PS[prfile][ti]

i++;

}

line++;

}

infile.close();

} else {

cout << "Error opening file ti.dat";

}

return status;

}

int MsApp::load_bp(){

int status = 0;

ifstream infile;

infile.open ("/home/arifhusen/ns2/ns-2.35/tcl/msprofiles/bp.dat", ifstream::in);

string str;

string bpx;

int line = 0;

if (infile.is_open()) {

while (getline(infile, str))

{

// cout << str << endl;

int i=0;

stringstream ssin(str);

while(ssin.good() && i <= 24){

ssin >> bpx;

int b = atoi(bpx.c_str());

BP[line][i] = b; // PS[prfile][bp start interval]

i++;

}

line++;

}

infile.close();

} else {

cout << "Error opening file ti.dat";

}

return status;

}

int MsApp::load_ps(){

int status = 0;

ifstream infile;

infile.open ("/home/arifhusen/ns2/ns-2.35/tcl/msprofiles/ps.dat", ifstream::in);

string str;

string psx;

int line = 0;

if (infile.is_open()) {

while (getline(infile, str))

{

// cout << str << endl;

int i=0;

stringstream ssin(str);

while(ssin.good() && i <= 24){

ssin >> psx;

int b = atoi(psx.c_str());

PS[line][i] = b; // PS[prfile][bp start interval]

i++;

}

line++;

}

infile.close();

} else {

cout << "Error opening file ti.dat";

}

return status;

}

int MsApp::get_pktSize(int bp, int prof) {

int pktsize = PS[trafprof_][bp];

//cout << pktsize << endl;

return pktsize;

}

int MsApp::get_bp(){

// this function will determin the current time and return the corresponding bp interval no defined in the bp.dat

double local_time = Scheduler::instance().clock();

int bp;

//cout << sizeof(BP) <<endl;

for (int i = 0; i <= 24; i++) {

int last_entry = 24;

if (local_time > BP[trafprof_][last_entry]){ // return the last bp

bp = last_entry;

//cout << local_time << ":" << BP[trafprof_][i] << endl;

} else {

if (local_time >= BP[trafprof_][i] && local_time <= BP[trafprof_][i+1])

{

bp = i;

//cout << local_time << ":" << BP[trafprof_][i] << endl;

}

}

}

//cout << local_time << ":" << bp << endl;

return bp;

}

**TIPS Header File**

/*

Author: Arif Husen , VU, MSCS Thesis Research

File: ibps.h

Written: 15/05/2016

Modifed: 15/05/2016

*/

#include <string.h>

#include "queue.h"

#include "address.h"

#include "config.h"

class Ibps : public Queue {

public:

Ibps();

protected:

void enque(Packet*);

Packet* deque();

Packet* ddq(int dq_t);

//int eenq(Packet *p , int enqto , int bp);

int get_enqto(int qSize);

PacketQueue *q0_; // First FIFO queue

PacketQueue *q1_; // First FIFO queue

PacketQueue *q2_; // Second FIFO queue

PacketQueue *q3_; // First FIFO queue

PacketQueue *q4_; // Second FIFO queue

//PacketQueue *q5_;

//PacketQueue *q6_;

int oprof3[4];

int oprof4[4];

int KF[5][5][5]; //int qsizes[level][qno][BP]

int qsizes[5][5][5]; //int qsizes[level][qno][BP]

int cfid;

int *a3;

int *a4;

int deq_turn_; // 1 for First queue 2 for Second

//int getQ_size(int BP , int qno);

int load_kfactors();

int load_qsizes();

//int getTIK_max(int max , int BP);

//int next_max(int max , int BP);

int* msort(int BP , int oprof[4]);

//int get_PIndex(int prof , double val);

int get_BP();

int getFid(int node, int dnode);

int dn_nodes_;

int load_ti();

int deqFactor;

int qu1;

int qu2;

int qu3;

int qu4;

int load_bp();

//int KF[3][5][4]; // KF[level][BP][queueno]

int profile0[5];

int profile1[5];

int profile2[5];

int profile3[5];

int profile4[5];

int profile5[5];

int BP0[5];

int BP1[5];

int BP2[5];

int BP3[5];

int BP4[5];

int BP5[5];

};

**TIPS Implementation File**

//

// Author: Arif Husen , MS Computer Sciences, Virtual University , Pakistan

// File: ibps.cc

// Written: 01/05/2016 (for ns-2.1b4a)

// Modifed: 01/05/2016 (for ns-2.1b8a)

//

#include "ibps.h"

#include <iostream>

#include <algorithm>

#include <packet.h>

#include <sstream>

#include <fstream>

#include <stdlib.h>

#include <cmath>

static class IbpsClass : public TclClass {

public:

IbpsClass() : TclClass("Queue/Ibps") {}

TclObject* create(int, const char*const*) {

return (new Ibps);

}

} class_ibps_round_robin;

Ibps::Ibps() {

q0_ = new PacketQueue;

q1_ = new PacketQueue;

q2_ = new PacketQueue;

q3_ = new PacketQueue;

q4_ = new PacketQueue;

//q6_ = new PacketQueue;

pq_ = q0_;

bind("dn_nodes_", &dn_nodes_);

deq_turn_ = 1;

qu1 = 0;

qu2 = 0;

qu3 = 0;

qu4 = 0;

load_ti();

load_bp();

load_kfactors();

load_qsizes();

//cout << qsizes[dn_nodes_][1][2] <<endl;

}

void Ibps::enque(Packet* p)

{

hdr_ip* iph = HDR_IP(p);

int bp = get_BP();

int enqto;

int n = 1;

//int fid;

//cout << qsizes[dn_nodes_][bp][1] << endl;

//cout << "hello" << endl;

////////// Handling the access nodes////////////////////////////////////////////////////

if (dn_nodes_ == 1) { // there is no downstream node , so just enqueue the packets in q1

q1_->enque(p);

if (q1_->length() > qsizes[dn_nodes_][1][bp]) {

q1_->remove(p);

drop(p);

}

}

////////// Handling the Core Nodes nodes////////////////////////////////////////////////////

if (dn_nodes_ == 3) { // there is no downstream node , so just enqueue the packets in q1

switch (iph->fid_) {

case 1 ... 4:

//cout << "hello" << endl;

enqto = get_enqto(qsizes[dn_nodes_][1][bp]);

if (enqto != 0) {

switch (enqto) {

case 1:

q1_->enque(p);

if (q1_->length() > qsizes[dn_nodes_][1][bp]) {

q1_->remove(p);

drop(p);

}

break;

case 2:

q2_->enque(p);

if (q2_->length() > qsizes[dn_nodes_][2][bp]) {

q2_->remove(p);

drop(p);

}

break;

case 3:

q3_->enque(p);

if (q3_->length() > qsizes[dn_nodes_][3][bp]) {

q3_->remove(p);

drop(p);

}

break;

}

}

break;

case 5:

enqto = get_enqto(qsizes[dn_nodes_][2][bp]);

if (enqto != 0) {

switch (enqto) {

case 1:

q1_->enque(p);

if (q1_->length() > qsizes[dn_nodes_][1][bp]) {

q1_->remove(p);

drop(p);

}

break;

case 2:

q2_->enque(p);

if (q2_->length() > qsizes[dn_nodes_][2][bp]) {

q2_->remove(p);

drop(p);

}

break;

case 3:

q3_->enque(p);

if (q3_->length() > qsizes[dn_nodes_][3][bp]) {

q3_->remove(p);

drop(p);

}

break;

}

}

break;

case 6:

enqto = get_enqto(qsizes[dn_nodes_][3][bp]);

if (enqto != 0) {

// eenq(p,enqto,bp);

switch (enqto) {

case 1:

q1_->enque(p);

if (q1_->length() > qsizes[dn_nodes_][1][bp]) {

q1_->remove(p);

drop(p);

}

break;

case 2:

q2_->enque(p);

if (q2_->length() > qsizes[dn_nodes_][2][bp]) {

q2_->remove(p);

drop(p);

}

break;

case 3:

q3_->enque(p);

if (q3_->length() > qsizes[dn_nodes_][3][bp]) {

q3_->remove(p);

drop(p);

}

break;

}

}

break;

}

}

if (dn_nodes_ == 4) {

switch (iph->fid_) {

case 1:

enqto = get_enqto(qsizes[dn_nodes_][1][bp]);

if (enqto != 0) {

//eenq(p,enqto,bp);

switch (enqto) {

case 1:

q1_->enque(p);

if (q1_->length() > qsizes[dn_nodes_][1][bp]) {

q1_->remove(p);

drop(p);

}

break;

case 2:

q2_->enque(p);

if (q2_->length() > qsizes[dn_nodes_][2][bp]) {

q2_->remove(p);

drop(p);

}

break;

case 3:

q3_->enque(p);

if (q3_->length() > qsizes[dn_nodes_][3][bp]) {

q3_->remove(p);

drop(p);

}

break;

case 4:

q4_->enque(p);

if (q4_->length() > qsizes[dn_nodes_][4][bp]) {

q4_->remove(p);

drop(p);

}

break;

}

}

break;

case 2:

enqto = get_enqto(qsizes[dn_nodes_][2][bp]);

if (enqto != 0) {

//eenq(p,enqto,bp);

switch (enqto) {

case 1:

q1_->enque(p);

if (q1_->length() > qsizes[dn_nodes_][1][bp]) {

q1_->remove(p);

drop(p);

}

break;

case 2:

q2_->enque(p);

if (q2_->length() > qsizes[dn_nodes_][2][bp]) {

q2_->remove(p);

drop(p);

}

break;

case 3:

q3_->enque(p);

if (q3_->length() > qsizes[dn_nodes_][3][bp]) {

q3_->remove(p);

drop(p);

}

break;

case 4:

q4_->enque(p);

if (q4_->length() > qsizes[dn_nodes_][4][bp]) {

q4_->remove(p);

drop(p);

}

break;

}

}

break;

case 3:

enqto = get_enqto(qsizes[dn_nodes_][3][bp]);

if (enqto != 0) {

//eenq(p,enqto,bp);

switch (enqto) {

case 1:

q1_->enque(p);

if (q1_->length() > qsizes[dn_nodes_][1][bp]) {

q1_->remove(p);

drop(p);

}

break;

case 2:

q2_->enque(p);

if (q2_->length() > qsizes[dn_nodes_][2][bp]) {

q2_->remove(p);

drop(p);

}

break;

case 3:

q3_->enque(p);

if (q3_->length() > qsizes[dn_nodes_][3][bp]) {

q3_->remove(p);

drop(p);

}

break;

case 4:

q4_->enque(p);

if (q4_->length() > qsizes[dn_nodes_][4][bp]) {

q4_->remove(p);

drop(p);

}

break;

}

}

break;

case 4:

enqto = get_enqto(qsizes[dn_nodes_][4][bp]);

if (enqto != 0) {

// eenq(p,enqto,bp);

switch (enqto) {

case 1:

q1_->enque(p);

if (q1_->length() > qsizes[dn_nodes_][1][bp]) {

q1_->remove(p);

drop(p);

}

break;

case 2:

q2_->enque(p);

if (q2_->length() > qsizes[dn_nodes_][2][bp]) {

q2_->remove(p);

drop(p);

}

break;

case 3:

q3_->enque(p);

if (q3_->length() > qsizes[dn_nodes_][3][bp]) {

q3_->remove(p);

drop(p);

}

break;

case 4:

q4_->enque(p);

if (q4_->length() > qsizes[dn_nodes_][4][bp]) {

q4_->remove(p);

drop(p);

}

break;

}

}

break;

} // end of switch

} // end f if

}

int Ibps::load_qsizes() {

int status = 1;

int sMulti = 1;

int k;

if (dn_nodes_ == 1) {

for (int q = 0; q<=4; q++) {

for (int bp = 0; bp <=4; bp++) {

k = sMulti * KF[dn_nodes_][q][bp];

if (q == 0) {qsizes[dn_nodes_][q][bp] = 0;} //becasue we are not using the q = 0

if (q == 1) {qsizes[dn_nodes_][q][bp] = k+profile0[bp];}

if (q == 2) {qsizes[dn_nodes_][q][bp] =0;}

if (q == 3) {qsizes[dn_nodes_][q][bp] = 0;}

if (q == 4) {qsizes[dn_nodes_][q][bp] = 0;} // because we are not using the q = 4

} //end of bp loop

} //end of q loop

}

if (dn_nodes_ == 3){

for (int q = 0; q<=4; q++) {

for (int bp = 0; bp<=4; bp++) {

k = sMulti * KF[dn_nodes_][q][bp];

if (q == 0) {qsizes[dn_nodes_][q][bp] = 0;} //becasue we are not using the q = 0

if (q == 1) {qsizes[dn_nodes_][q][bp] = k+(profile0[bp]+profile1[bp]+profile2[bp]+profile3[bp]);}

if (q == 2) {qsizes[dn_nodes_][q][bp] = k+profile4[bp];}

if (q == 3) {qsizes[dn_nodes_][q][bp] = k+profile5[bp];}

if (q == 4) {qsizes[dn_nodes_][q][bp] = 0;} // because we are not using the q = 4

} //end of bp loop

} //end of q loop

}

if (dn_nodes_ == 4){

for (int q = 0; q<=4; q++) {

for (int bp = 0; bp <=4; bp++) {

k = sMulti * KF[dn_nodes_][q][bp];

if (q == 0) {qsizes[dn_nodes_][q][bp] = 0;} //becasue we are not using the q = 0

if (q == 1) {qsizes[dn_nodes_][q][bp] = k+profile0[bp];}

if (q == 2) {qsizes[dn_nodes_][q][bp] = k+profile1[bp];}

if (q == 3) {qsizes[dn_nodes_][q][bp] = k+profile2[bp];}

if (q == 4) {qsizes[dn_nodes_][q][bp] = k+profile3[bp];} // because we are not using the q = 4

} //end of bp loop

} //end of q loop

}

return status;

}

Packet* Ibps::deque(){

Packet *p;

int BP;

BP = get_BP();

//-------------------------- Handle All Control Messages------------------------------------------

if (pq_->length() > 0 ) {

pq_->deque();

}

//-------------------------- Handle Access Nodes------------------------------------------

if (dn_nodes_ == 1) {

p = ddq(1);

}

//-------------------------- Handle Aggregation Nodes------------------------------------------

if (dn_nodes_ == 4){

switch (deq_turn_) {

case 1:

p = ddq(1);

qu1++;

if(p == 0) {

p = ddq(2);

qu2++;

deq_turn_ = 3;

} else {

//cout << "q2 len....." << q2_->length() <<":" << p << endl;

if (((KF[dn_nodes_][1][BP]-qu1) <= 0)) {deq_turn_ = 2; qu1 = 0;} else { deq_turn_ = 1;}

//cout << deq_turn_ <<endl;

//deq_turn_ = 1;

}

break;

case 2:

//cout << "case 2" <<endl;

p = ddq(2);

qu2++;

if(p == 0) {

p = ddq(3);

qu3++;

deq_turn_ = 4;

} else {

//deq_turn_ = 2;

if ((KF[dn_nodes_][2][BP]-qu2) <= 0) {deq_turn_ = 3; qu2= 0;} else { deq_turn_ = 2;}

}

break;

case 3:

//cout << "case 3" <<endl;

p = ddq(3);

qu3++;

if(p == 0) {

p = ddq(4);

qu4++;

deq_turn_ = 1;

} else {

//deq_turn_ = 3;

if ((KF[dn_nodes_][3][BP]-qu3) <= 0) {deq_turn_ = 4; qu3= 0;} else { deq_turn_ = 3;}

}

break;

case 4:

//cout << "case 4" <<endl;

p = ddq(4);

qu4++;

if(p == 0) {

p = ddq(1);

qu1++;

deq_turn_ = 2;

} else {

//deq_turn_ = 4;

if ((KF[dn_nodes_][4][BP]-qu4) <= 0) {deq_turn_ = 1;qu4= 0;} else { deq_turn_ = 4;}

}

break;

}

}

//-------------------------- handle core nodes------------------------------------------

if (dn_nodes_ == 3) {

switch (deq_turn_) {

case 1:

p = ddq(1);

qu1++;

if(p == 0) {

p = ddq(2);

qu2++;

deq_turn_ = 3;

} else {

if ((KF[dn_nodes_][1][BP] - qu1) <= 0) {deq_turn_ = 2; qu1 = 0;} else { deq_turn_ = 1;}

}

break;

case 2:

p = ddq(2);

qu2++;

if(p == 0) {

p = ddq(3);

qu3++;

deq_turn_ = 1;

} else {

if ((KF[dn_nodes_][2][BP]-qu2) <= 0) {deq_turn_ = 3; qu2 = 0;} else { deq_turn_ = 2;}

}

break;

case 3:

p = ddq(3);

qu3++;

if(p == 0) {

p = ddq(1);

qu1++;

deq_turn_ = 2;

} else {

if ((KF[dn_nodes_][3][BP]-qu3) <= 0) {deq_turn_ = 1; qu3 = 0;} else { deq_turn_ = 3;}

}

break;

}

}

return (p);

}

Packet* Ibps::ddq(int dq_t) {

Packet* p;

int bp = get_BP();

//int k = 2;

int cqsize; // current queue size

if (dn_nodes_ == 1) {

p = q1_->deque();

}

if (dn_nodes_ == 4) {

switch(dq_t) {

case 1:

cqsize = q1_->length();

if (cqsize > KF[dn_nodes_][1][bp]) {

for (int i=1; i <= KF[dn_nodes_][1][bp]; i++) {

p = q1_->deque();

}

} else {p = q1_->deque();}

break;

case 2:

cqsize = q2_->length();

if (cqsize > KF[dn_nodes_][2][bp]) {

for (int i=1; i <= KF[dn_nodes_][2][bp]; i++) {

p = q2_->deque();

}

}else { p = q2_->deque();}

break;

case 3:

cqsize = q3_->length();

if (cqsize > KF[dn_nodes_][3][bp]) {

for (int i=1; i <= KF[dn_nodes_][3][bp]; i++) {

p = q3_->deque();

}

}{ p = q3_->deque();}

break;

case 4:

cqsize = q4_->length();

if (cqsize > KF[dn_nodes_][4][bp]) {

for (int i=1; i <= KF[dn_nodes_][4][bp]; i++) {

p = q4_->deque();

}

}{ p = q4_->deque();}

break;

}

}

if (dn_nodes_ == 3) {

switch(dq_t) {

case 1:

cqsize = q1_->length();

if (cqsize > KF[dn_nodes_][1][bp]) {

for (int i=1; i <=KF[dn_nodes_][1][bp]; i++) {

p = q1_->deque();

}

} else { p = q1_->deque(); }

break;

case 2:

cqsize = q2_->length();

if (cqsize > KF[dn_nodes_][2][bp]) {

for (int i=1; i <= KF[dn_nodes_][2][bp]; i++) {

p = q2_->deque();

}

}else { p = q2_->deque();}

break;

case 3:

cqsize = q3_->length();

if (cqsize > KF[dn_nodes_][3][bp]) {

for (int i=1; i <= KF[dn_nodes_][3][bp]; i++) {

p = q3_->deque();

}

}{ p = q3_->deque();}

break;

}

}

return (p);

}

int* Ibps::msort(int BP , int oprof[4]){

int TI_q1;

int TI_q2;

int TI_q3;

int TI_q4;

if (dn_nodes_ == 1 || dn_nodes_ == 4){

// get the values of respected BP

TI_q1 = profile0[BP];

TI_q2 = profile1[BP];

TI_q3 = profile2[BP];

TI_q4 = profile3[BP];

int q_serving_order[4]; // TI values of all queus

q_serving_order[0] = TI_q1;

q_serving_order[1] = TI_q2;

q_serving_order[2] = TI_q3;

q_serving_order[3] = TI_q4;

std::sort(q_serving_order, q_serving_order + 4,std::greater<int>()); // sort the queue values

//Now get the queue numbers in above order

int q1 = 0;

int q2 = 0;

int q3 = 0;

int q4 = 0;

// determine the first queu position ( Assume that all TIs are integer and different from each other)

if (profile0[BP] == q_serving_order[0]) { q1 = 1;}

if (profile1[BP] == q_serving_order[0]) { q1 = 2;}

if (profile2[BP] == q_serving_order[0]) { q1 = 3;}

if (profile3[BP] == q_serving_order[0]) { q1 = 4;}

if (profile0[BP] == q_serving_order[1]) { q2 = 1;}

if (profile1[BP] == q_serving_order[1]) { q2 = 2;}

if (profile2[BP] == q_serving_order[1]) { q2 = 3;}

if (profile3[BP] == q_serving_order[1]) { q2 = 4;}

if (profile0[BP] == q_serving_order[2]) { q3 = 1;}

if (profile1[BP] == q_serving_order[2]) { q3 = 2;}

if (profile2[BP] == q_serving_order[2]) { q3 = 3;}

if (profile3[BP] == q_serving_order[2]) { q3 = 4;}

if (profile0[BP] == q_serving_order[3]) { q4 = 1;}

if (profile1[BP] == q_serving_order[3]) { q4 = 2;}

if (profile2[BP] == q_serving_order[3]) { q4 = 3;}

if (profile3[BP] == q_serving_order[3]) { q4 = 4;}

// prepare arrary to return

oprof[0] = q1;

oprof[1] = q2;

oprof[2] = q3;

oprof[3] = q4;

}

if (dn_nodes_ == 3){

// get the values of respected BP

TI_q1 = profile0[BP]+profile1[BP]+profile2[BP]+profile3[BP];

TI_q2 = profile4[BP];

TI_q3 = profile5[BP];

TI_q4 = 0;

int q_serving_order[4]; // TI values of all queus

q_serving_order[0] = TI_q1;

q_serving_order[1] = TI_q2;

q_serving_order[2] = TI_q3;

q_serving_order[3] = TI_q4;

sort(q_serving_order, q_serving_order + 4,greater<int>()); // sort the queue values

//Now get the queue numbers in above order

int q1 = 0;

int q2 = 0;

int q3 = 0;

int q4 = 0;

// determine the first queu position ( Assume that all TIs are integer and different from each other)

int agg_ti_prof = profile0[BP]+profile1[BP]+profile2[BP]+profile3[BP];

if (agg_ti_prof == q_serving_order[0]) { q1 = 1;}

if (profile4[BP] == q_serving_order[0]) { q1 = 2;}

if (profile5[BP] == q_serving_order[0]) { q1 = 3;}

//if (profile3[BP] == q_serving_order[0]) { q1 = 4;}

if (agg_ti_prof == q_serving_order[1]) { q2 = 1;}

if (profile4[BP] == q_serving_order[1]) { q2 = 2;}

if (profile5[BP] == q_serving_order[1]) { q2 = 3;}

//if (profile3[BP] == q_serving_order[1]) { q2 = 4;}

if (agg_ti_prof == q_serving_order[2]) { q3 = 1;}

if (profile4[BP] == q_serving_order[2]) { q3 = 2;}

if (profile5[BP] == q_serving_order[2]) { q3 = 3;}

//if (profile3[BP] == q_serving_order[2]) { q3 = 4;}

if (agg_ti_prof== q_serving_order[3]) { q4 = 1;}

if (profile4[BP] == q_serving_order[3]) { q4 = 2;}

if (profile5[BP] == q_serving_order[3]) { q4 = 3;}

//if (profile3[BP] == q_serving_order[3]) { q4 = 4;}

// prepare arrary to return

oprof[0] = q1;

oprof[1] = q2;

oprof[2] = q3;

oprof[3] = 0;

}

return oprof;

}

int Ibps::get_BP() {

double ts = Scheduler::instance().clock();

int BP;

if (ts >= 0 && ts <= 2) { BP = 0; }

if (ts > 2 && ts <= 4) { BP = 1; }

if (ts > 4 && ts <= 6) { BP = 2; }

if (ts > 6 && ts <= 8) { BP = 3; }

if (ts > 8) { BP = 4; }

return BP;

}

int Ibps::load_ti(){

//double ts = Scheduler::instance().clock();

//cout << ts << endl;

int status = 0;

ifstream infile;

infile.open ("/home/arifhusen/ns2/ns-2.35/tcl/msprofiles/ti.dat", ifstream::in);

string str;

string tix;

int line = 0;

if (infile.is_open()) {

while (getline(infile, str))

{

//cout << str << endl;

int i=0;

stringstream ssin(str);

while(ssin.good() && i <= 5){

if (line == 0) { ssin >> tix; int b = atoi(tix.c_str()); profile0[i] = b; }

if (line == 1) { ssin >> tix; int b = atoi(tix.c_str()); profile1[i] = b; }

if (line == 2) { ssin >> tix; int b = atoi(tix.c_str()); profile2[i] = b; }

if (line == 3) { ssin >> tix; int b = atoi(tix.c_str()); profile3[i] = b; }

if (line == 4) { ssin >> tix; int b = atoi(tix.c_str()); profile4[i] = b; }

if (line == 5) { ssin >> tix; int b = atoi(tix.c_str()); profile5[i] = b; }

//cout << i <<endl;

i++;

}

line++;

}

infile.close();

} else {

cout << "Error opening file ti.dat";

}

return status;

}

int Ibps::load_bp(){

int status = 0;

ifstream infile;

infile.open ("/home/arifhusen/ns2/ns-2.35/tcl/msprofiles/bp.dat", ifstream::in);

string str;

string bpx;

int line = 0;

if (infile.is_open()) {

while (getline(infile, str))

{

// cout << str << endl;

int i=0;

stringstream ssin(str);

while(ssin.good() && i < 6){

if (line == 0) { ssin >> bpx; int b = atoi(bpx.c_str()); BP0[i] = b; }

if (line == 1) { ssin >> bpx; int b = atoi(bpx.c_str()); BP1[i] = b; }

if (line == 2) { ssin >> bpx; int b = atoi(bpx.c_str()); BP2[i] = b; }

if (line == 3) { ssin >> bpx; int b = atoi(bpx.c_str()); BP3[i] = b; }

if (line == 4) { ssin >> bpx; int b = atoi(bpx.c_str()); BP4[i] = b; }

// cout << BP0[i] <<endl;

i++;

}

line++;

}

infile.close();

} else {

cout << "Error opening file ti.dat";

}

return status;

}

int Ibps::get_enqto(int qSize) {

int bp = get_BP();

int enqto = 0;

if (dn_nodes_ == 3) {

//a3 = msort(bp,oprof3); // this will return the requested profile in descending order , so that we assing the values

enqto = 1;

}

if (dn_nodes_ == 3) {

a3 = msort(bp,oprof3); // this will return the requested profile in descending order , so that we assing the values

for (int i = 0; i<=4; i++){

if (qSize == qsizes[dn_nodes_][a3[i]][bp]) { enqto = i+1;}

}

}

if (dn_nodes_ == 4) {

a4 = msort(bp,oprof4); // this will return the requested profile in descending order , so that we assing the values

for (int i = 0; i<=4; i++){

if (qSize == qsizes[dn_nodes_][a4[i]][bp]) { enqto = i+1;}

}

}

//cout << enqto << endl;

return enqto;

}

int Ibps::load_kfactors() {

int status = 1;

int MinQ_no;

int mnqno;

int min_ti;

//int KF[3][5][5]; //int qsizes[level][qno][BP]

int kfmul = 1;

// for level one all queus have the factor 1

if (dn_nodes_ == 1) {

for (int q = 0; q<=4; q++) {

for (int bp = 0; bp <=4; bp++) {

KF[dn_nodes_][q][bp] = 1;

}

}

//kf = 1;

}

if (dn_nodes_ == 4) {

int oprf4[4];

int *b4;

int min_q_inBP4[5];

//int MinQ_no;

//int min_ti;

b4 = msort(0,oprf4); mnqno = b4[2]; min_q_inBP4[0] = mnqno;

b4 = msort(1,oprf4); mnqno = b4[2]; min_q_inBP4[1] = mnqno;

b4 = msort(2,oprf4); mnqno = b4[2]; min_q_inBP4[2] = mnqno;

b4 = msort(3,oprf4); mnqno = b4[2]; min_q_inBP4[3] = mnqno;

b4 = msort(4,oprf4); mnqno = b4[2]; min_q_inBP4[4] = mnqno;

for (int q =0; q<=4; q++) {

for (int bp = 0; bp <=4; bp++) {

//int oprf4[4];

//int *b4;

MinQ_no = min_q_inBP4[bp];

if (MinQ_no == 1) { min_ti = profile0[bp]; }

if (MinQ_no == 2) { min_ti = profile1[bp]; }

if (MinQ_no == 3) { min_ti = profile2[bp]; }

if (MinQ_no == 4) { min_ti = profile3[bp]; }

if (min_ti == 0) { min_ti = 1;}

if (q == 0) {KF[dn_nodes_][q][bp] = kfmul+1; }

if (q == 1 && profile0[bp] > 0) {KF[dn_nodes_][q][bp] = kfmul+((profile0[bp] / min_ti)+1); }

if (q == 2 && profile1[bp] > 0) {KF[dn_nodes_][q][bp] = kfmul+((profile1[bp] / min_ti)+1);}

if (q == 3 && profile2[bp] > 0) {KF[dn_nodes_][q][bp] = kfmul+((profile2[bp] / min_ti)+1); }

if (q == 4 && profile3[bp] > 0) {KF[dn_nodes_][q][bp] = kfmul+((profile3[bp] / min_ti)+1); }

//KF[dn_nodes_][q][bp] = 1;

}

}

}

if (dn_nodes_ == 3) {

//minq[bp] = qno;

int oprf3[4];

int *b3;

int min_q_inBP3[5];

//int MinQ_no;

//int min_ti;

b3 = msort(0,oprf3); mnqno = b3[2]; min_q_inBP3[0] = mnqno;

b3 = msort(1,oprf3); mnqno = b3[2]; min_q_inBP3[1] = mnqno;

b3 = msort(2,oprf3); mnqno = b3[2]; min_q_inBP3[2] = mnqno;

b3 = msort(3,oprf3); mnqno = b3[2]; min_q_inBP3[3] = mnqno;

b3 = msort(4,oprf3); mnqno = b3[2]; min_q_inBP3[4] = mnqno;

for (int q = 0; q<=4; q++) {

//cout << q <<endl;

for (int bp = 0; bp <=4; bp++) {

MinQ_no = min_q_inBP3[bp];

if (MinQ_no == 1) { min_ti = (profile0[bp]+profile1[bp]+profile2[bp]+profile3[bp]); }

if (MinQ_no == 2) { min_ti = profile4[bp]; }

if (MinQ_no == 3) { min_ti = profile5[bp]; }

if (min_ti == 0) { min_ti = 1;}

if (q == 0) {KF[dn_nodes_][q][bp] = 0;}

if (q == 1) {KF[dn_nodes_][q][bp] = kfmul+(((profile0[bp]+profile1[bp]+profile2[bp]+profile3[bp])/min_ti)+1); }

if (q == 2 && profile4[bp] > 0) {KF[dn_nodes_][q][bp] = kfmul+(((profile4[bp]) / min_ti)+1);}

if (q == 3 && profile4[bp] > 0) {KF[dn_nodes_][q][bp] = kfmul+(((profile5[bp]) / min_ti)+1);}

}

}

}

return status;

}
