# Supplementary figures and images for: Improving scheduling performance in congested networks
Source: PeerJ Comput Sci. 2021 Nov 1;7:e754. doi: 10.7717/peerj-cs.754 (PMC8576556; doi:10.7717/peerj-cs.754)

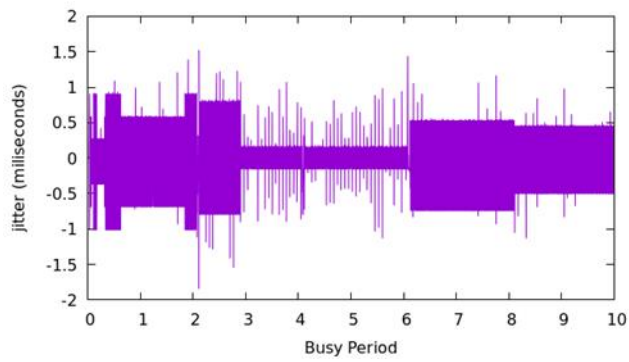

(a) - Node-1

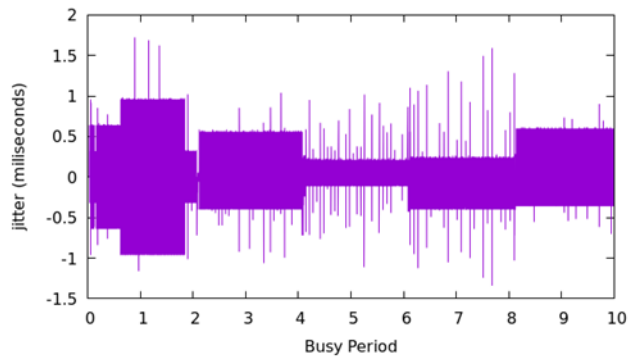

(b) - Node-2

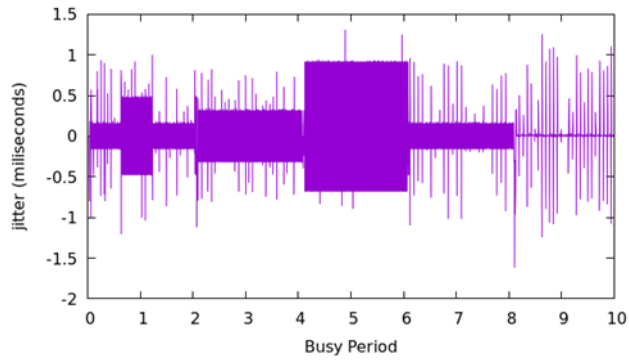

(c) - Node-3

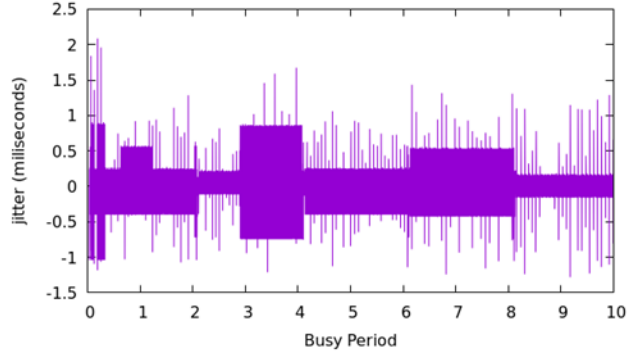

(d) - Node-4

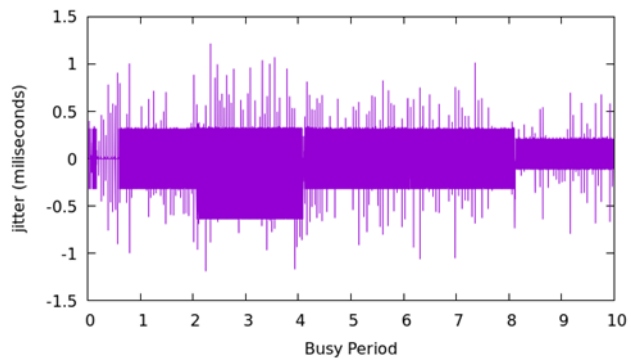

(e) - Node-5

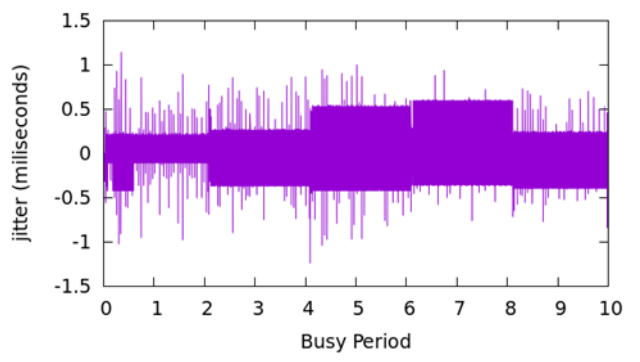

(f) - Node-6

Supplement: Supplemental Information 3 [file peerj-cs-07-754-s003.pdf]

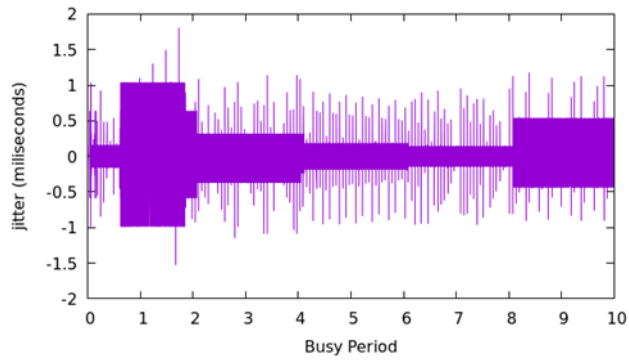

(a) : Node-1

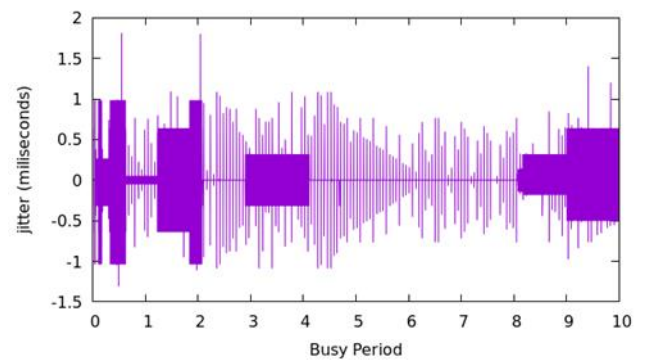

(b) : Node-2

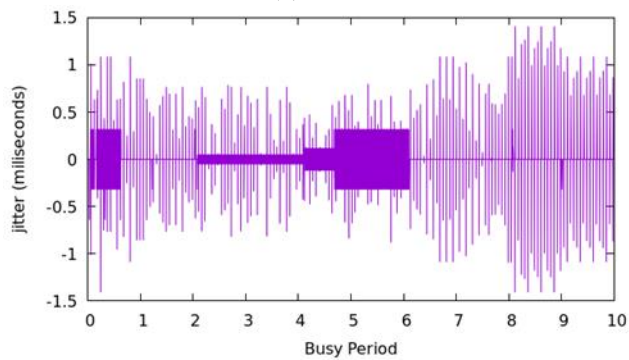

(c) : Node-2

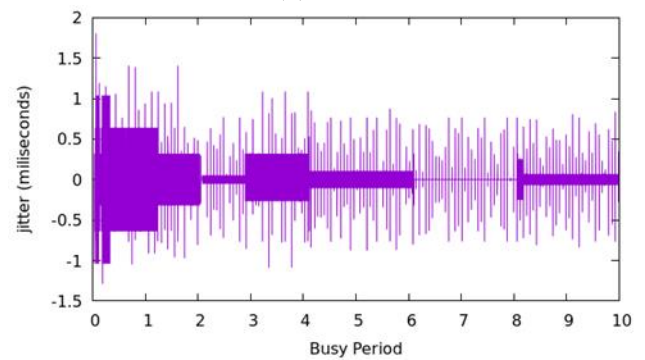

(d) : Node-2

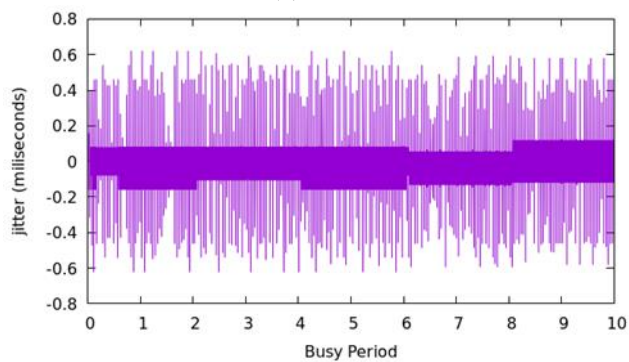

(e) : Node-2

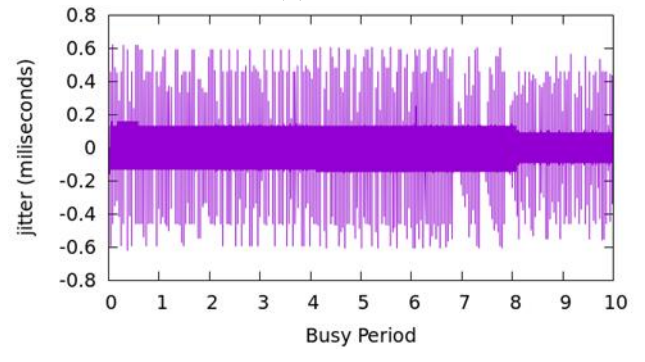

(f) : Node-2

Supplement: Supplemental Information 4 [file peerj-cs-07-754-s004.pdf]
